# Candidate drugs associated with sensitivity of cancer cell lines with *DLST* amplification or high mRNA levels

**DOI:** 10.18632/oncotarget.28342

**Published:** 2023-01-12

**Authors:** Christina Kuhn, Myriam Boeschen, Manuel Philip, Torsten Schöneberg, Doreen Thor, Susanne Horn

**Affiliations:** ^1^Rudolf Schönheimer Institute of Biochemistry, Medical Faculty, University of Leipzig, Leipzig 04103, Germany; ^2^Institute of Pathology, Medical Faculty, University of Leipzig, Leipzig 04103, Germany; ^3^Department of Dermatology, University Hospital Essen, University Duisburg-Essen, and German Cancer Consortium (DKTK) partner site Essen/Düsseldorf, Essen 45122, Germany; ^*^These authors contributed equally to this work

**Keywords:** neuroblastoma, drug sensitivity, drug resistance, drug repurposing, DLST

## Abstract

Overexpression of the dihydrolipoamide S-succinyltransferase (*DLST*) is associated with poor outcome in neuroblastoma patients and triple-negative breast cancer (TNBC) and specifically with the oxidative phosphorylation (OXPHOS) pathway. Inhibitors of OXPHOS were previously suggested as a potential therapeutic strategy for a subset of patients with high-risk neuroblastoma. Here, we tested if cell lines with *DLST* amplifications or high mRNA levels were associated with sensitivity to 250 drugs from the Genomics of Drug Sensitivity in Cancer (GDSC) dataset by comparing them to cell lines without these changes. *DLST*-altered cell lines were more sensitive to 7 approved drugs, among these obatoclax mesylate, a BCL2 inhibitor that reduces OXPHOS in human leukemia stem cells. Moreover, several protein kinase inhibitors were identified to be efficient in cell lines with *DLST* amplifications or high mRNA levels, suggesting a vulnerability of *DLST*-altered cell lines for drugs targeting the ERK/MAPK pathway. Furthermore, increased *DLST* expression in cell lines with driver mutations in *KRAS* supported this relationship. We therefore conclude that, in addition to OXPHOS, protein kinases could be potential targets of therapy in the presence of *DLST* amplifications or high mRNA levels. The new drug candidates proposed here could serve in experimental testing on drug efficacy in knock-in cell lines and *DLST*-activated tumors.

## INTRODUCTION

Recently, an increased expression of the dihydrolipoamide S-succinyltransferase (*DLST*) gene has been shown to predict poor outcome of neuroblastoma and triple-negative breast cancer (TNBC) [1, 2]. In contrast, knockdown of *DLST* led to apoptosis in T-cell acute lymphoblastic leukemia and in TNBC [1, 3] suggesting a *DLST* dependence for tumor cell viability and, consequently highlighting *DLST* as a potential target for cancer therapy. With respect to its cellular function, DLST converts α-ketoglutarate to succinyl-CoA during oxidative decarboxylation and produces NADH for the oxidative phosphorylation (OXPHOS). Specifically, DLST is an E2 subcomponent of the mitochondrial oxoglutarate dehydrogenase complex (OGDC), catalyzing the overall conversion of 2-oxoglutarate to succinyl-CoA during the tricarboxylic acid (TCA) cycle. There is evidence that certain cancer cells use the TCA cycle extensively for energy production, offering potential therapeutic opportunities by targeting the TCA cycle in cancer cells [4]. In line with this, *DLST*-overexpressing cell lines showed increased OXPHOS gene signatures [2]. The OXPHOS inhibitor IACS-010759 targets the electron transport chain and leads to a reduction in tumor weight, size, and proliferation of neuroblastoma cells in zebrafish and mouse xenografts [2]. Other FDA-approved drugs, used to treat non-oncologic indications such as metformin, arsenic trioxide, and atovaquone, have also been identified as OXPHOS inhibitors *in vivo* [5]. Thus, additional vulnerabilities of *DLST-*activated tumors within and beyond OXPHOS inhibition and drug repurposing are conceivable.

Therefore, we set out to identify new candidates for the treatment of *DLST-*activated tumors. With the advent of complex genetic datasets of roughly 1000 cell lines in the Cancer Cell Line Encyclopedia (CCLE) and on drug resistance in the Genomics of Drug Sensitivity in Cancer project (GDSC), analyses of drug sensitivity have become possible on a larger scale [6, 7]. We took advantage of these data to test if a potential *DLST* activation, caused by overexpression or copy number amplification, is associated with drug sensitivity and whether additional OXPHOS inhibitors may serve as therapeutics for tumors with high levels of *DLST*.

## RESULTS

To identify drugs that inhibit viability of specifically *DLST*-activated tumor cells, we compared cell lines with supposedly activating changes of *DLST* (DNA amplification, high mRNA levels) to cell lines without *DLST* changes. In the light of the described *DLST* activation in neuroblastoma [2], we aimed at detecting an effect specific to 70 cancer cell lines derived from the nervous system. However, only one of these had an increased *DLST* copy number and none of them showed high mRNA levels, hence, a test of nervous system tumors was not possible (Supplementary Table 1).

**Figure 1:**
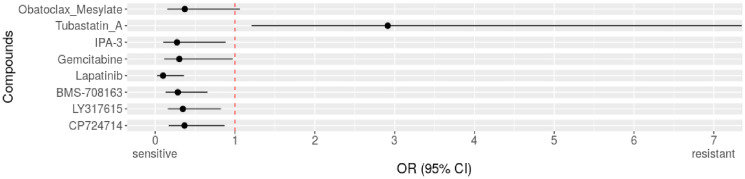
Identified drugs for *DLST*-activated cell lines across cancer types. Compared to *DLST* wild types across entities. Forest plot with odds ratio (OR) for resistance of altered cell lines to drugs. OR>1 indicates resistance, OR<1 indicates sensitivity.

Therefore, we extended our analysis across all entities available, of which 55 of 872 (6.31%) cell lines showed these supposedly activating genetic alterations in *DLST*. We tested resistance and sensitivity for the full set of 250 individual drugs and found that *DLST-*activated cell lines were more often sensitive to 7 drugs compared to control cell lines without such changes (OR<1, Figure 1, Supplementary Tables 1–3). In Table 1 all drugs associated with sensitivity together with their function and current applications of these drugs in neuroblastoma and other cancers are given. In this literature research we found that the drugs lapatinib and gemcitabine were approved, also as combination therapy, in various cancer types, such as breast, gastric, and lung cancer. Others showed promising anticancer activity *in vivo* (LY317615, CP724714, and obatoclax mesylate) and *in vitro* (IPA3, BMS708163) (Table 1). Neuroblastoma tumor size was also reduced in mouse models for gemcitabine and the drug combination of lapatinib and YM155. Under obatoclax mesylate cell lines derived from neuroblastoma showed significantly decreased apoptosis. We next analyzed which compounds had been previously involved in processes related to OXPHOS and found only obatoclax mesylate to reduce OXPHOS in leukemia stem cells. Therefore, we aimed at identifying the signaling pathway involvement of the remaining 6 identified compounds and found 4 out of 6 were protein kinase inhibitors of the extracellular signal-regulated kinases/mitogen-activated protein kinase (ERK/MAPK) signaling pathway: lapatinib, IPA3, LY317615, and CP724714. Of note, we tested 250 individual drugs and after correcting for multiple testing (Bonferroni method), none of the drugs were significantly associated with sensitivity of *DLST*-activated cell lines.

**Table 1:** Drugs associated with sensitivity of *DLST*-activated cell lines and their relation to OXPHOS and ERK/MAPK pathway

Drug (*p*-value)	OR	Mut: s (%), r	Wt: s (%), r	Function	Current applications in cancer types	Related to OXPHOS; ERK/MAPK pathway
Lapatinib (*p* = 0.0005)	0.1003 (0.02391–0.3629)	7 (63.64%), 4	28 (14.51%), 165	inhibition of EGFR and HER2 if high ERB2 expression	drug combination (i.e., lapatinib plus YM155) decreased neuroblastoma tumor size in an *in vivo* model [18], approved in combination with capecitabine in patients with advanced HER2-positive breast cancer [19]	lapatinib increases ROS levels in models that are sensitive to erbB1/2 blockade [20]; upstream activator of ERK/MAPK pathway [11]
IPA3 (*p* = 0.0263)	0.2742 (0.1024–0.883)	5 (13.88%), 31	21 (4.30%), 467	inhibitor of Pak1	melanoma and colon carcinoma cell lines that carry mutations in NRAS and KRAS are more sensitive to IPA3 [21]	downregulation of Pak2 associated with a shift toward OXPHOS, PAK1 knockout resulted in increased glycolysis [22]; upstream activator of ERK/MAPK pathway [13]
BMS 708163 (*p* = 0.0034) also: Avagacestat	0.2844 (0.1319–0.6562)	10 (28.57%), 25	52 (10.25%), 455	γ-secretase inhibitor of Aβ40 and Aβ42	tested in lung cancer after resistance to gefitinib, induced high level of apoptosis [23]	no relevance found in OXPHOS; no relevance found in ERK/MAPK pathway
Gemcitabine (*p* = 0.0363)	0.3033 (0.1144–0.9706)	5 (13.88%), 31	23 (4.74%), 462	standard chemotherapy	Neuroblastoma is highly sensitive to gemcitabine tested (*in vitro* and *in vivo*) [24], approved in non-small cell lung cancer, pancreatic, bladder, and breast cancer [25]	treated PDAC cells with phenformin (MRC I inhibitor) + standard chemotherapy (gemcitabine), this treatment is synergistic specifically in high OXPHOS cells [26]; no relevance found in ERK/MAPK pathway
LY317615 (*p* = 0.0142) also: Enzastaurin	0.3476 (0.1594–0.8258)	9 (25%), 27	53 (10.45%), 454	PKCβ (protein kinase C-β) selective inhibitor	most promising in B-cell lymphomas [27], could be effective in acute myeloid leukemia therapy [14]	no relevance found in OXPHOS; suppressor of ERK [14]
CP724714 (*p* = 0.0276)	0.368 (0.1594–0.8258)	9 (24.32%), 28	54 (10.65%), 453	ErbB2 (HER2) inhibitor	has been tested for the treatment of ErbB2-positive malignancies [28], induces G1 cell cycle block in erbB2-overexpressing human breast carcinoma cells [29]	exacerbated OXPHOS dependency frequently characterizes resistance against chemotherapy or tyrosine kinase inhibitors [30]; upstream activator of ERK/MAPK pathway [12]
Obatoclax Mesylate (*p* = 0.0484)	0.3721 (0.1524–1.061)	6 (16.67%), 30	34 (7.02%) 450	inhibitor of the Bcl-2 family of proteins (conserved family that share BH domains)	obatoclax significantly increased apoptosis and autophagy in neuroblastoma cells [31], obatoclax-induced apoptosis was associated with leukemic cell differentiation [32]	inhibition of BCL2 reduce OXPHOS in human leukemia stem cells [8]; no relevance found in ERK/MAPK pathway

OR for resistance (>1: resistance, <1: sensitivity, lower and upper 95% confidence intervals). Numbers of and sensitive (s) and resistant (r) cell lines (and % sensitive) for *DLST*-activated (Mut) and wild type (Wt) cell lines, respectively. Drug function and role in OXPHOS and applications in neuroblastoma and other cancer types. Abbreviations: PDAC: Pancreatic ductal adenocarcinoma; OR: Odds ratio; MRC: mitochondrial respiratory complex.

We hypothesized that in an inverse scenario of the described associations above, drugs associated with sensitivity in *DLST-*activated cell lines would be associated with resistance in *DLST-*deactivated cell lines (33 of 872, 3.78%). This assumption was true for lapatinib, CP724714, and obatoclax mesylate, which showed an OR for resistance >1 in cell lines with *DLST* deactivation, however, lacking statistical significance (Supplementary Table 4).

**Figure 2:**
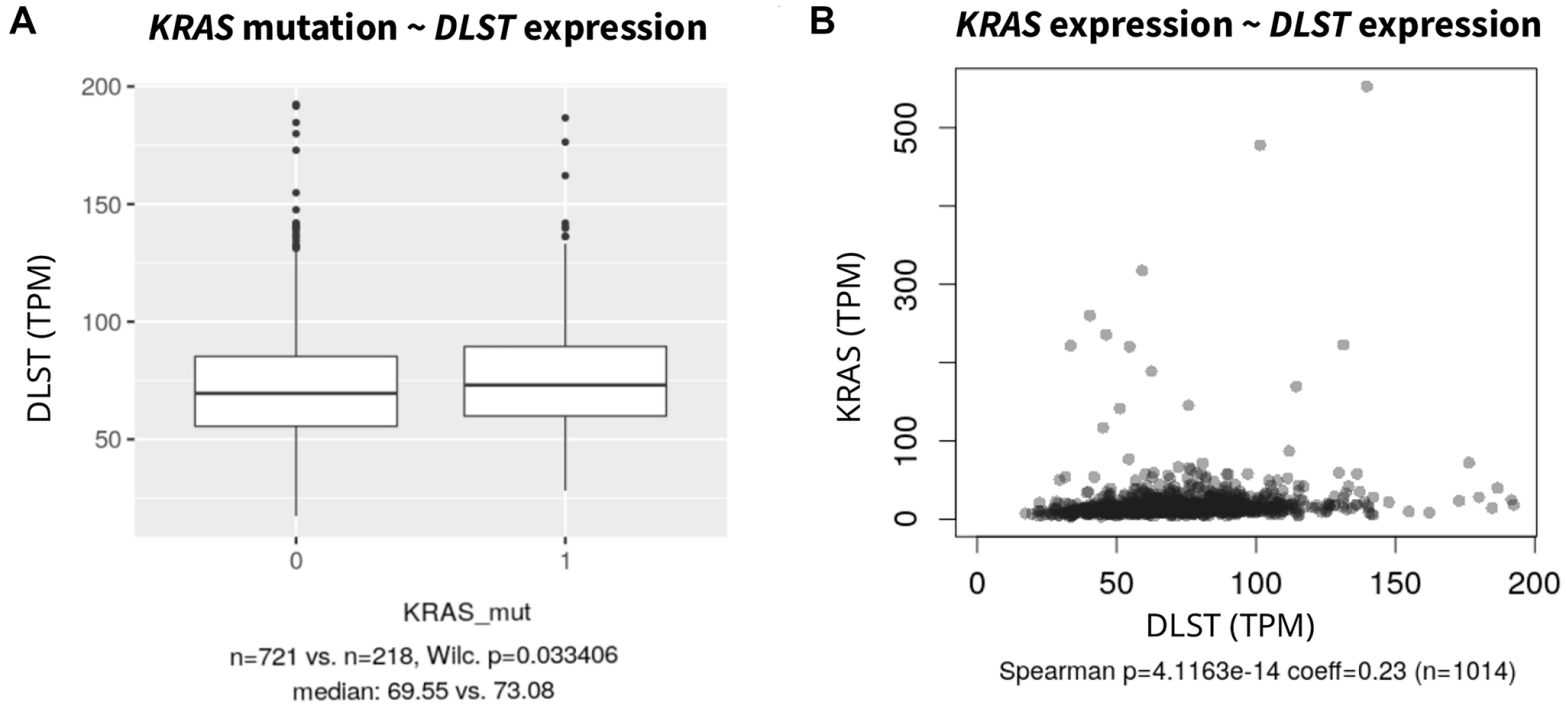
(**A**) Association of *DLST* expression in cell lines with *KRAS* driver mutations against *KRAS* wild type and (**B**) Spearman correlation with *KRAS* expression (without one outlier; *KRAS* expression >1500). For other common driver mutations such as *NRAS*, *HRAS* and *BRAF* see Supplementary Figure 1.

As stated above, the primary analyses identified only one OXPHOS inhibitor but several protein kinase inhibitors were associated with sensitivity. Because this points to an involvement of DLST in the ERK/MAPK pathway, we asked if the sensitivity of *DLST*-activated cell lines to MAPK inhibitors could be due to concomitant changes in the ERK/MAPK pathway. Such common driver mutations occur in *NRAS*, *KRAS* and *HRAS*. However, *DLST*-activated cell lines did not show alterations in any of the *RAS* genes more frequently than all other samples (OR = 1.24, *p* = 0.23). We, therefore, investigated whether *DLST* expression was associated with mutation or expression of common genes involved in the ERK/MAPK pathway. *DLST* expression was not associated with driver mutations in *BRAFV600* (*p* = 0.856), *HRAS* (*p* = 0.791) or *NRAS* (*p* = 0.609) (Supplementary Figure 1). However, cell lines with driver mutations in *KRAS* had higher *DLST* expression than controls (*p* = 0.033, Figure 2A). Also, coexpression of *DLST* and *ERBB2* (Spearman coef = 0.08, *p* = 8.46e-3), *HRAS* (Spearman coef = 0.08, *p* = 6.10e-3), *KRAS* (Spearman coef = 0.16, *p* = 1.25e-7, Figure 2B), and *PAK1* (Spearman coef = 0.09, *p* = 3.5e-3) was observed.

## DISCUSSION

New therapy options are still being explored for the treatment of neuroblastoma including potential molecular pathways and genetic aberrations. While our results indicate that *DLST* amplification or high mRNA levels are rather rare in cell lines derived from the nervous system, they may nevertheless have an impact on cancer growth as *DLST* overexpression had led to increased tumor burden and higher frequency of disseminated disease in a zebrafish model of MYCN-driven neuroblastoma [2]. Moreover, beyond this entity, high *DLST* expression was associated with poor overall survival also in triple-negative breast cancer patients [1] and may warrant studying of *DLST* activation in further cancer entities.

Recently, the elevated expression of *DLST* in cancer was associated with aggressiveness in neuroblastoma and with the OXPHOS pathway specifically. Studies showed that OXPHOS can be upregulated to support the growth, survival, and migration of cancer cells, and upregulated DLST expression may pose a mechanism to increase OXPHOS [2, 5]. Given that OXPHOS inhibition slowed tumor growth in zebrafish and mouse xenografts of neuroblastoma cells [2], OXPHOS inhibitors could , therefore, constitute a potential vulnerability of *DLST-*altered tumors. Three OXPHOS inhibitors (antimycin, oligomycin, IACS-010759) were suggested to inhibit growth of neuroblastoma cells and partly induced apoptosis in zebrafish and mouse xenografts of high-risk neuroblastoma [2]. In the light of a putative association of *DLST*-activated tumors with elevated OXPHOS, it is of particular interest that in our analysis of a large number of cancer cell lines, *DLST*-activated cell lines were sensitive to obatoclax mesylate more often than control cell lines. This inhibitor of the Bcl-2-family proteins has previously shown to reduce OXPHOS in human leukemia stem cells [8]. Moreover, besides the candidate drug obatoclax mesylate, other BCL-2 inhibitors, which were not included in the GDSD dataset, such as venetoclax and navitoclax showed promising results in neuroblastoma-derived cell lines and tumors [9]. These data suggest that obatoclax mesylate and other Bcl-2 inhibitors could be efficient in tumors with *DLST-*activating alterations and should be further investigated in this context [9].

**Figure 3:**
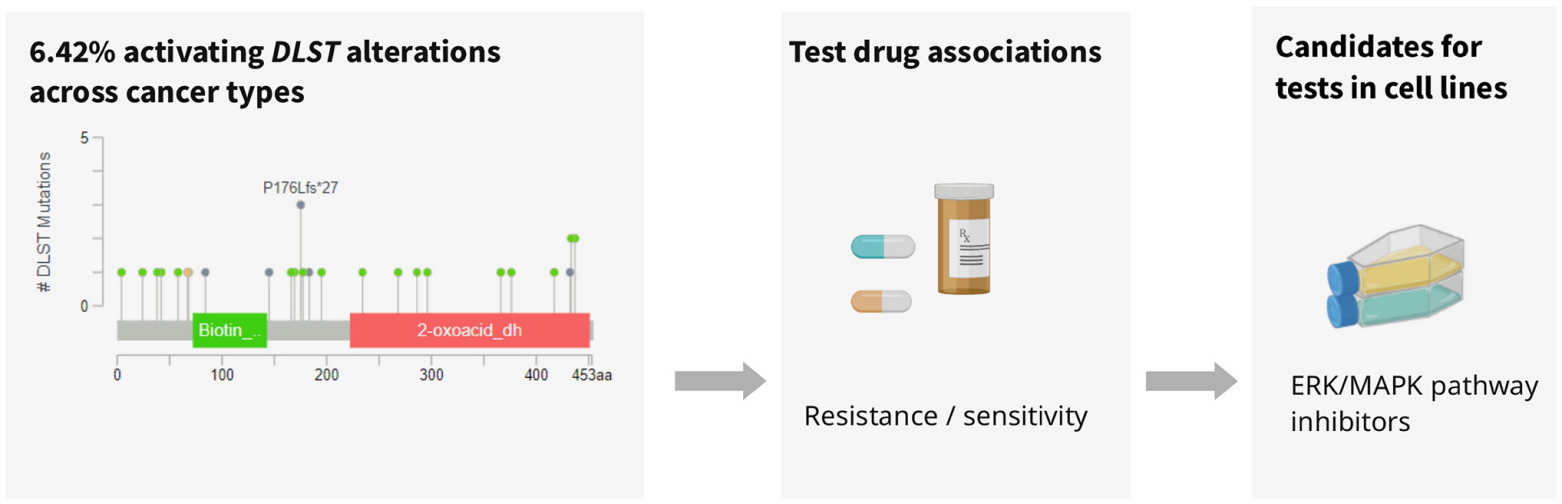
Analysis of *DLST*-activated cell lines revealed sensitivity to protein kinase inhibiting the ERK/MAPK pathway.

In addition to the described role of *DLST* in OXPHOS and the identified OXPHOS inhibitor, we found a group of protein kinase inhibitors potentially suitable for therapy of such tumors (Figure 3). Protein kinase inhibitors are used for efficient cancer therapy to modulate cell proliferation, survival, and transformation [10]. All protein kinase inhibitors presented here target the ERK/MAPK pathway, a signaling pathway that is aberrantly activated in cancer. Lapatinib and CP724714 reduce ERK/MAPK pathway activity via inhibition of the upstream epidermal growth factor receptor HER2/ERBB2 [11, 12]. IPA3 binds to the inactive PAK group I molecule, which can also activate the ERK/MAPK signaling pathway [13] and LY317615 suppresses the activation of ERK [14]. As in general, the ERK/MAPK pathway activation is a frequent event in cancer, we asked whether the sensitivity of *DLST*-activated cell lines to MAPK inhibitors could be due concomitant changes in the ERK/MAPK pathway. Indeed, there was an increased *DLST* expression in cell lines with *KRAS* mutations (Figure 3) as well as co-expression of *DLST* and several genes involved in the ERK/MAPK pathway, which may explain the observed sensitivity to inhibitors of the ERK/MAPK pathway. Furthermore, a link between DLST and ERK/MAPK signaling pathways was previously suggested, as circDLST was shown to sponge with microRNA, activating the NRAS/MEK/ERK signaling in gastric cancer [15]. Thus, *DLST* potentially constitutes a proxy for ERK/MAPK pathway activation. Our data provides a potential starting point for future research in understanding the role of *DLST* in cancer and the power of ERK/MAPK-inhibitors in *DLST*-activated cancer. These findings further highlight how understanding the underlying molecular networks can help to achieve rational care. Initially, the Notch inhibitor BMS 708163 was not linked to either the OXPHOS or MAPK/ERK pathway. However, in agreement with our results, Anderson et al. recently described that cell lines with overexpressed MYC, which is transcriptionally regulated by Notch, have the ability to stabilize DLST [3].

The observed associations reported here have to be seen in the context of the manifold genetic changes that cancer cell lines have accumulated. However, the pooling of hundreds of cell lines in groups may ameliorate the confounding effects of individual genetic variation to some extent and may thus allow more general, mechanistic insights. Nevertheless, the proposed candidate drugs could be subject to further rigorous tests using knock-out and knock-in experiments of cell lines, xenografts in animal models or association studies of *DLST*-levels in tumors and patient outcome to determine the relevance of *DLST* in cancer and its potential utility in predictions of clinical outcome.

## MATERIALS AND METHODS

To test differences in drug sensitivity in *DLST* genetically and expressionally altered cancer cell lines, we used genetic data of 1,739 cell lines in the Cancer Cell Line Encyclopedia dataset (CCLE, accessed 06/2022 at https://www.cbioportal.org/study/summary?id=ccle_broad_2019, Supplementary Table 5). We integrated these genetic data with drug resistance data on 250 drugs of the Genomics of Drug Sensitivity in Cancer project GDSC [6, 7]. Combining the drug resistance data from the GDSC database with the genetic data from the CCLE samples at https://tools.hornlab.org/GDSC/ [16], resulted in a dataset of 872 cell lines. The documented various types of genetic changes in the CCLE dataset provide information on copy number variation, mRNA levels, point mutations, and gene fusions. Here, we focused on supposedly activating changes of *DLST* defined by copy number amplification or high mRNA expression of *DLST* (Supplementary Tables 1–3) because, as part of the mitochondrial oxoglutarate dehydrogenase complex (OGDC) and rate limiting enzyme of the TCA cycle, we assumed *DLST* levels to have direct impact on OGDC activity. We then also analyzed supposedly deactivating changes defined as homozygous deletion, low mRNA, frameshift or nonsense variants (Supplementary Tables 6, 7, 4). Low and high mRNA expression levels were defined with a minimum z-score of -2 and 2, respectively, based on RNA-seq RPKM units compared to the expression distribution of diploid samples in cBioPortal [17]. To determine whether the defined group of cell lines was rather resistant or sensitive to the tested drugs, we calculated odds ratios (OR) for resistance by comparing the counts of resistant and sensitive cell lines in the genetically changed (*DLST*-activated) and the control cell line group without any *DLST* changes (*DLST* wild types) (two-sided Fisher’s exact and chi-square tests). Bonferroni corrected *p*-value thresholds were < 0.0023 testing 22 pathways (each pathway containing pooled sets of drugs assigned to this pathway) and *p* < 0.0002 testing 250 individual drugs.

Initially, we analyzed all cell lines across entities and then specifically a subgroup of 70 cell lines that had been documented to derive from the central nervous system (*n* = 54) or from neuroblastoma (autonomous ganglia, *n* = 16, Supplementary Table 1). We further tested *DLST* expression in cell lines across entities (CCLE 2019 cohort) with *BRAFV600, NRAS*, *KRAS*, and *HRAS* driver mutations and tested co-expression with genes involved in the ERK/MAPK pathway (*ERBB2, HRAS, NRAS, KRAS, PAK1,* via cBioPortal).

## SUPPLEMENTARY MATERIALS

















## Abbreviations

BCL: B-cell lymphoma
CCLE: cancer cell line encyclopedia
DLST: dihydrolipoamide S-succinyltransferase
EGFR: epidermal growth factor receptor
ERBB2: erb-b2 receptor tyrosine kinase 2
ERK: extracellular signal-regulated kinase
FDA: food and drug administration
GDSC: genomics of drug sensitivity in cancer
HER2: human epidermal growth factor 2
IC: half-maximal inhibitory concentration
MAPK: mitogen activated protein kinase
MRC: mitochondrial respiratory complex
MYCN: N-myc proto-oncogene
OGDC: oxoglutarate dehydrogenase complex
OR: odds ratio
OXPHOS: oxidative phosphorylation
PAK1: P21 activated kinase 1
PDAC: Pancreatic ductal adenocarcinoma
RPKM: reads per kilobase per million
TCA: the citric acid cycle
TNBC: triple-negative breast cancer
TPM: Transcripts per million
