# Supplementary material for: Candidate drugs associated with sensitivity of cancer cell lines with DLST amplification or high mRNA levels
Source: Oncotarget. 2023 Jan 12;14:14–20. doi: 10.18632/oncotarget.28342 (PMC9836382; doi:10.18632/oncotarget.28342)
Supplement: Supplementary file 1 [file oncotarget-14-28342-s001.pdf]

Candidate drugs associated with sensitivity of cancer cell lines with *DLST* amplification or high mRNA levels

SUPPLEMENTARY MATERIALS

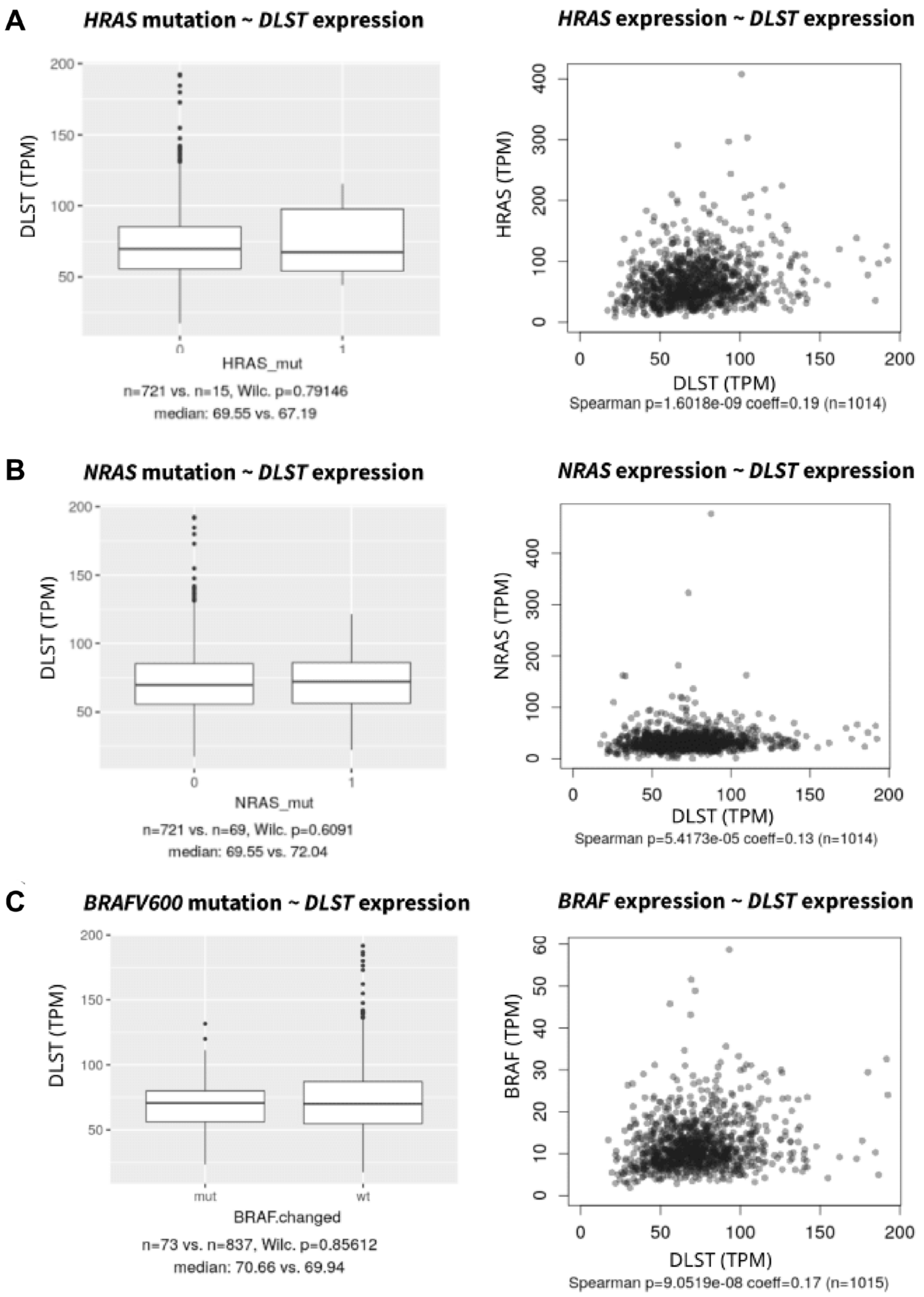

Supplementary Figure 1: Associations of *DLST* expression with (**A**) HRAS, (**B**) NRAS and (**C**) BRAF driver mutations and corresponding gene expression levels.

Supplementary Table 1: *DLST*-activating alterations across CCLE samples obtained February 2022. See Supplementary Table 1

Supplementary Table 2: Counts of resistant and sensitive cell lines from drug test of *DLST*-activating alterations across CCLE samples across cancer entities obtained February 2022. See Supplementary Table 2

Supplementary Table 3: Odds ratios from drug test of *DLST*-activating alterations across CCLE samples across cancer entities obtained February 2022. See Supplementary Table 3

Supplementary Table 4: Odds ratios from drug test of *DLST*-deactivating alterations across CCLE samples across cancer entities obtained February 2022. See Supplementary Table 4

Supplementary Table 5: *DLST* genetically and expressionally altered cancer cell lines obtained from the Cancer Cell Line Encyclopedia dataset. See Supplementary Table 5

Supplementary Table 6: *DLST*-deactivating alterations across CCLE samples obtained February 2022. See Supplementary Table 6

Supplementary Table 7: Counts of resistant and sensitive cell lines from drug test of *DLST*-deactivating alterations across CCLE samples across cancer entities obtained February 2022. See Supplementary Table 7
